# Using neutrophil to lymphocyte ratio to predict discharge among geriatric patients with influenza infection in emergency department

**DOI:** 10.1097/MD.0000000000030261

**Published:** 2022-08-26

**Authors:** Jing-Cheng Jheng, Yen-Ting Tseng, Te-Hao Wang, Li-Fu Chen, Jui-Yuan Chung

**Affiliations:** a Department of Emergency Medicine, National Yang-Ming University Hospital, I-Lan, Taiwan; b School of Medicine, National Yang-Ming University, Taipei, Taiwan; c Department of Emergency Medicine, Cathay General Hospital, Taipei, Taiwan; d School of Medicine, Fu Jen Catholic University, Taipei, Taiwan; e School of Medicine, National Tsing Hua University, Hsinchu, Taiwan.

**Keywords:** emergency department, geriatric, influenza, neutrophil to lymphocyte ratio

## Abstract

The neutrophil-to-lymphocyte ratio (NLR) is used to predict the prognosis of various diseases, such as coronavirus disease 2019, community-acquired pneumonia, bacteremia, and endocarditis. However, NLR has never been reported to predict patient discharge in geriatric patients with influenza infection. This retrospective case-control study enrolled geriatric patients (≥65 years) with influenza virus infection who visited the emergency department of a medical center between January 01, 2010 and December 31, 2015. Demographic data, vital signs, past histories, influenza subtypes, outcomes, and disposition were analyzed. The optimal NLR cut-off value to predict patient discharge was determined using the Youden index. We also evaluated the accuracy of NLR in predicting patient discharge using logistic regression and receiver operating characteristic analysis. The study included 409 geriatric patients in the emergency department with a mean age of 79.5 years and an approximately equal sex ratio. NLR was significantly lower in the discharged group than in the nondischarged group (5.8 ± 3.7 vs 9.7 ± 8.4). Logistic regression revealed that patients with NLR ≤ 6.5 predicted discharge with an odds ratio of 3.62. The Hosmer–Lemeshow goodness-of-fit test was calculated as 0.36, and the adjusted area under the receiver operating characteristic was 0.75. The negative predictive value of NLR ≤ 6.5, to predict patient discharge, was 91.8%. NLR ≤ 6.5 is a simple and easy-to-obtain laboratory tool to guide the physicians to discharge geriatric patients with influenza infection in the crowded emergency department.

## 1. Introduction

There are approximately 5 million cases of severe complicated influenza illness during the seasonal influenza pandemic, causing over 290,000 to 650,000 deaths worldwide annually.^[1,2]^ The elderly are vulnerable to influenza infection and are more likely to have serious complications,^[3]^ as in the United States, more than 90 percent of influenza-related deaths occur in the elderly.^[4]^ Furthermore, approximately 44,000 cases of influenza-related hospitalization were correlated with patients aged ≥ 65 years.^[5]^

The neutrophil-to-lymphocyte ratio (NLR) is an effective laboratory biomarker that indicates a systemic inflammatory response,^[6]^ as neutrophils are a crucial component of the innate immune system, whereas lymphocytes represent the adaptive immune system. Neutrophils secrete large amounts of cytokines and chemokines to regulate immune responses.^[7]^ During severe influenza infection, substantial amounts of cytokines and chemokines are released due to the overactivation of the immune system,^[7]^ ultimately resulting in fatal outcomes. In contrast, low levels of lymphocytes have been observed during severe novel influenza A infection.^[8]^

NLR is also correlated with severe diseases, including acute pancreatitis, liver disease, rheumatic diseases, and acute respiratory distress syndrome.^[9–14]^ Furthermore, NLR is useful in identifying hospitalized patients with fever due to infection and those with fever due to noninfectious causes.^[15–17]^ Finally, NLR has also been proven to predict the prognosis of different types of infections, such as community-acquired pneumonia, bacteremia, and endocarditis.^[18,19]^

Geriatric patients tend to be vulnerable and have multiple comorbidities. The decision to discharge or admit such patients, especially discharge, may be difficult, as emergency department (ED) physicians must consider several pressures such as the context of time-based targets, ED flow, and resource allocation.^[20]^ Therefore, a compact strategy to discharge geriatric patients with influenza infection during the flu pandemic may be crucial. Clinical prediction tools, such as geriatric influenza death (GID) score, are available to evaluate the prognosis of geriatric patients with influenza infection using physiological parameters and laboratory statistics and further suggest patient disposition based on the mortality risk determined by the score.^[21]^ As neutrophil and lymphocyte counts may reflect the intensity of the immune response during an infection episode in adult patients, it may further indicate disease severity and eventually be used as a guide for physicians in patient disposition, especially discharge. To our knowledge, NLR to predict patient discharge in geriatric patients with influenza infection has never been reported; therefore, we conducted this study to delineate this issue.

## 2. Methods

### 2.1. Study design, setting, and participants

This study was conducted in the ED of the Cathay General Hospital, which consists of 800 ward beds and 40 ED beds. The annual number of ED visiting patients was approximately 55,000, with 33% of elderly adult patients.^[22,23]^ Patients who visited the ED between January 1, 2010 and December 31, 2015, fulfilling the following criteria will be enrolled in the study: age ≥ 65 years; tympanic membrane temperature ≥ 37.2°C or an increase in baseline tympanic membrane temperature ≥ 1.3°C^[22,23]^; and influenza infection, defined as a positive pharyngeal or throat swab test, using antigen detection.^[24]^

### 2.2. Definition of variables and primary outcome

The primary outcome of this study was patient discharge from the ED. Discharge was defined as patients discharged directly from the ED within 4 hours without being admitted to the ward or intensive care unit and revisited within 72 hours (based on clinical judgement, including physical examinations, vital signs, laboratory data, general appearance, etc, by the board-certified emergency physicians). The 30-days mortality was defined as geriatric patients with influenza infection who died within the 30-days telephone follow-up process. A prolonged hospital stay was defined as >9 days.^[25,26]^ Intensive care unit (ICU) admission was defined according to the critical care management committee of the Cathay General Hospital with the following criteria: patients who required advanced respiratory support; patients who encountered shock or circulatory failure; 2 or more of the organ systems required support; and (4) physician’s clinical judgement.^[27]^ Systemic inflammatory response syndrome (SIRS) criteria were defined as follows: heart rate >90 beats per minute; respiratory rate >20 breaths per minute; temperature <36°C or >38°C, white blood cell count <4000/mm^3^ or >12,000/mm^3^; and band form >10%.^[28]^ Evidence of pneumonia is defined as new pulmonary infiltration visible on the chest image; while evidence of urinary tract infection is defined as pyuria noted from the urine analysis. The SIRS criteria score will be obtained and calculated while the patients arrive at the ED. For discharged patients, telephone follow-up was performed to obtain 30-days survival.

### 2.3. Data collection and assignment to case and control groups

A medical chart review was performed on geriatric patients who fulfilled the criteria of this study. The included patients were divided into “discharged” and “nondischarged groups.” Demographic characteristics were also acquired, including age, vital signs (obtained at the triage), past medical history, laboratory data, disease severity, ICU admission, and influenza subtype. Required data that are not recorded in the patient’s medical chart will be considered as missing data, and the patient will be excluded.

### 2.4. Ethical statement

This study was approved by the Institutional Review Board of Cathay General Hospital and was conducted in accordance with the Declaration of Helsinki. Informed consent from the participants was waived because of the retrospective design of this study.

### 2.5. Statistical analysis

G-power 3.0 was used to calculate the power of this study size (409 patients), which was 0.80. Statistical analysis was performed using Statistical Package for the Social Sciences (SPSS) 23.0 for Mac (SPSS Inc., Chicago, IL). Normally distributed continuous data were presented as means ± standard deviation (SD), while data that were not normally distributed were presented as median (interquartile range). Continuous data of the independent samples were compared using the *t*-test or Mann–Whitney–Wilcoxon test, whereas categorical variables were compared using the Pearson chi-square test or Fisher exact test. The Youden index was used to identify the optimal cut-off point of NLR to predict patient discharge. The area under the receiver operating characteristic curve (AUROC) was used to evaluate the ability of the NLR to discriminate patient discharge. The AUROCs were adjusted for potential confounding factors. Sensitivity, specificity, positive predictive value, and negative predictive value (NPV) were calculated.

## 3. Results

**Figure 1.**
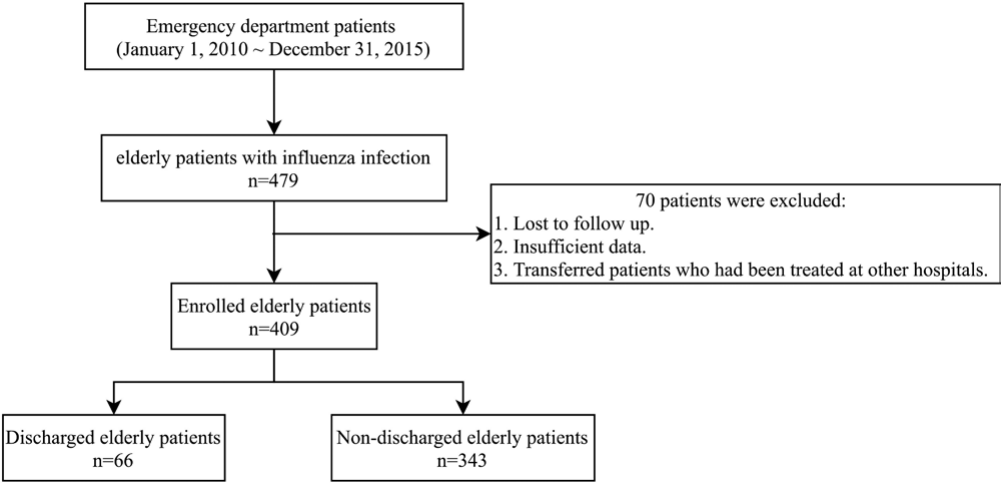
Flowchart of this study.

A total of 409 patients were finally included (Fig. 1), after excluding 70 patients who were lost to follow-up, had insufficient data, or transferred patients who had been treated at other hospitals. The male-to-female ratio was approximately equal, while the mean ± SD of age was significantly lower in the discharged group (75.4 ± 7.2) than in the nondischarged group (80.4 ± 8.4). Furthermore, more young, elderly adults aged 65 to 74 years were noted in the discharged group (47.0%) than in the nondischarged group (27.4%), whereas younger elderly adults aged > 85 years were noted in the discharged group (10.0%) than in the nondischarged group (30.0%) (Table 1).

**Table 1:** Characteristics of discharged and nondischarged patients among geriatric patients with influenza infection in the emergency department.

Characteristics	Total patients (n = 409)	Discharged (n = 66)	Non-discharged (n = 343)	*P* value
Age, yr	79.5 ± 8.3	75.4 ± 7.2	80.4 ± 8.4	<.01
Age subgroup				
Young elderly (65–74 yr)	30.6	47.0	27.4	<.01
Moderately elderly (75–84 yr)	42.5	42.4	42.6	.98
Old elderly (≥85 yr)	26.9	10.6	30.0	<.01
Male, sex	50.1	45.5	51.0	.41
Vital signs				
GCS	13.9 ± 2.32	14.8 ± 1.0	13.8 ± 2.5	<.01
SBP (mm Hg)	146.1 ± 30.5	153.4 ± 26.1	144.7 ± 31.1	.03
DBP (mm Hg)	74.2 ± 17.4	77.6 ± 13.9	73.6 ± 17.9	.04
Heart rate (/min)	98.8 ± 20.5	96.5 ± 14.9	99.3 ± 21.5	.33
Respiratory rate (/min)	21.2 ± 4.1	19.0 ± 1.8	21.7 ± 4.3	<.01
Tympanic temperature (°C)	38.1 ± 0.9	38.3 ± 0.8	38.1 ± 0.9	.81
Past history				
Hypertension	64.3	43.9	68.2	<.01
Diabetes	39.8	30.3	41.1	.22
COPD	27.1	4.5	19.8	.01
Coronary artery disease	25.1	9.1	25.4	.01
Stroke	15.8	10.6	16.9	.20
Cancer	14.9	12.1	15.5	.48
Congestive heart failure	9.0	10.5	1.5	.02
Dementia	2.2	3.0	2.2	.67
Laboratory data				
WBC (cells/mm^3^)	10,590.0 ± 5820.0	8009.5 ± 2687.0	11,068.9 ± 6050.2	<.01
Neutrophil	8101.9 ± 4446.9	6006.6 ± 2560.1	8481.3 ± 4609.5	<.01
Lymphocyte	1377.2 ± 1205.7	1187.2 ± 497.1	1411.5 ± 949.4	.06
NLR	9.1 ± 7.2	5.8 ± 3.7	9.7 ± 8.4	<.01
Influenza subtypes				
Influenza A	68.0	71.2	67.3	.54
Influenza B	29.3	28,8	29.4	.91
Influenza A + B	2.7	0.0	3.2	.14
Influenza vaccination	23.2	68.2	14.6	<.01
Severity				
SIRS	1.9 ± 1.1	1.4 ± 1.0	2.0 ± 1.1	<.01
30-day mortality rate	4.9	0.0	5.8	.04

Data were presented as % or mean ± SD.

DBP = diastolic blood pressure, ED = Emergency Department, GCS = Glasgow coma scale, NLR = neutrophil to lymphocyte ratio, SBP = systolic blood pressure, SD = standard deviation, SIRS = systemic inflammatory response syndrome, WBC = white blood cell count.

Geriatric patients with influenza infection had more unstable vital signs in the nondischarged group than in the discharged group. In addition, the mean ± SD of the respiratory rate was significantly higher in the nondischarged group (21.7 ± 4.3 per minute and 19.0 ± 1.8/ per minute) than in the discharged group (20.8 ± 3.4/ per minute). Meanwhile, the Glasgow coma scale and systolic blood pressure were both lower in the nondischarged group (13.8 ± 2.5 and 144.7 ± 31.1) (Table 1).

Comorbidities such as hypertension, coronary artery disease (CAD), and chronic obstructive pulmonary disease (COPD) were significantly greater in the nondischarged group (68.2%, 19.8%, and 25.4%) than in the discharged group (43.9%, 4.5%, and 9.1%), respectively. For the laboratory data, white blood cell count and neutrophil cell count were significantly higher in the nondischarged group (11,068.9 ± 6050.2 and 8481.3 ± 4609.5) than in the discharged group (8009.5 ± 2687.0 and 6006.6 ± 2560.1), respectively. In addition, lymphocyte count was higher in the nondischarged group (1411.5 ± 949.4), although the difference was not statistically significant (*P* = .06). Consequently, NLR was higher in the nondischarged group (9.7 ± 8.4) than in the discharged group (5.8 ± 3.7) (Table 1).

For influenza subtypes, both the percentages of influenza A and B infections in the discharged and nondischarged groups showed no significant difference. Once the influenza infection was confirmed, the patients were treated with either oseltamivir or zanamivir within 24 hours. The influenza vaccination percentage was higher in the discharged group (68.2%) than in the nondischarged group (14.6%). In addition, the SIRS criteria were higher in the nondischarged group (2.0 ± 1.1), while the 30-days mortality rate was 5.8% in the nondischarged group and none in the discharged group (Table 1).

Higher neutrophil counts were noted in the SIRS ≧ 2 group (10,415.9 ± 5184.9) than in the SIRS < 2 group (7056.8 ± 3623.1), with a *P*-value < .01. A trend of higher neutrophil counts and lymphocyte counts was noted in the mortality group (8018.7 ± 5782.6 and 1988.9 ± 922.9), hospital stay > 9 days group (8965.1 ± 4977.9 and 1473.4 ± 1211.6), and ICU group (9763.8 ± 5578.9 and 1637.6 ± 1711.3) than in the survivor group (5338.6 ± 3409.4 and 1110.2 ± 583.2), hospital stay < 9 days group (7323.7 ± 3751 and 1270.4 ± 1202.8), and non-ICU group (7888 ± 4243.0 and 1343.8 ± 1117.1). These results also consequence in higher NLR in the 3 groups at 8.9 ± 6.6 (*P* value < 0.95), 10.6 ± 8.9, (*P* value < .01), and 10.6 ± 8.8 (*P* value = .05). Although higher NLR was noted in the evidence of pneumonia and urinary tract infection group for 10.0 ± 7.9 (compared to 8.9 ± 7.9 in the no evidence of pneumonia and urinary tract infection group), no statistically significant difference was noted between these 2 groups, with *P*-value .22 (Table 2).

**Table 2:** Comparison of neutrophil count, lymphocyte count, and NLR between mortality, hospital stay > 9 days and hospital stay < 9 days, ICU and non-ICU, SIRS ≥ 2 and SIRS < 2, evidence of pneumonia or urinary tract infection and no evidence of pneumonia or urinary tract infection.

	Mortality (n = 20)	Survivor (n = 389)	*P* value
Neutrophil	8018.7 ± 5782.6	5338.6 ± 3409.4	.98
Lymphocyte	1988.9 ± 922.9	1110.2 ± 583.2	.11
NLR	8.9 ± 6.6	5.2 ± 3.2	.95
	**Hospital stay** > **9 d (n = 192**)	**Hospital stay < 9 d (n = 217**)	***P* value**
Neutrophil	8965.1 ± 4977.9	7323.7 ± 3751	<.01
Lymphocyte	1473.4 ± 1211.6	1270.4 ± 1202.8	.26
NLR	10.6 ± 8.9	7.7 ± 6.6	<.01
	**ICU (n = 46**)	**Non-ICU (n = 363**)	***P* value**
Neutrophil	9763.8 ± 5578.9	7888 ± 4243.0	.15
Lymphocyte	1637.6 ± 1711.3	1343.8 ± 1117.1	.30
NLR	10.6 ± 8.8	8.9 ± 7.8	.05
	**SIRS ≥ 2 (n = 258**)	**SIRS < 2 (n = 151**)	***P* value**
Neutrophil	9120.7 ± 4847.0	6086.2 ± 2938.3	<.01
Lymphocyte	1453.6 ± 1179.2	1211.2 ± 814.2	.19
NLR	10.5 ± 8.9	6.6 ± 4.8	<.01
	Evidence of pneumonia or urinary tract infection* **(n = 308**)	**No evidence of pneumonia or urinary tract infection (n = 105**)	***P* value**
Neutrophil	9224.9 ± 4237.7	7803.6 ± 4459.8	.01
Lymphocyte	1293.3 ± 1060.6	1399.5 ± 1057.3	.63
NLR	10.0 ± 7.9	8.9 ± 7.9	.22

ICU = intensive care unit, NLR = neutrophil-to-lymphocyte ratio, SIRS = systemic inflammatory response syndrome.

* Evidence of pneumonia is defined as new pulmonary infiltration visible on the chest image; evidence of urinary tract infection is defined as pyuria noted form the urine analysis.

The optimal cut-off point for NLR to predict discharge among elderly patients with influenza infection was calculated as ≤6.5, based on the Youden index. Logistic regression showed that NLR ≤ 6.5 predicted patient discharge with an odds ratio of 3.62 (Table 3). The Hosmer-Lemeshow goodness-of-fit was 0.36 for NLR ≤ 6.5.

**Table 3:** Logistic regression analysis of NLR ≤ 6.5 to predict discharge in geriatric patients with influenza infection.

	*B*	Odds ratio	95% CI	*P* value
NLR ≤ 6.5	1.29	3.62	2.0–6.5	<.01

CI = confidence interval, NLR = neutrophil to lymphocyte ratio.

**Figure 2.**
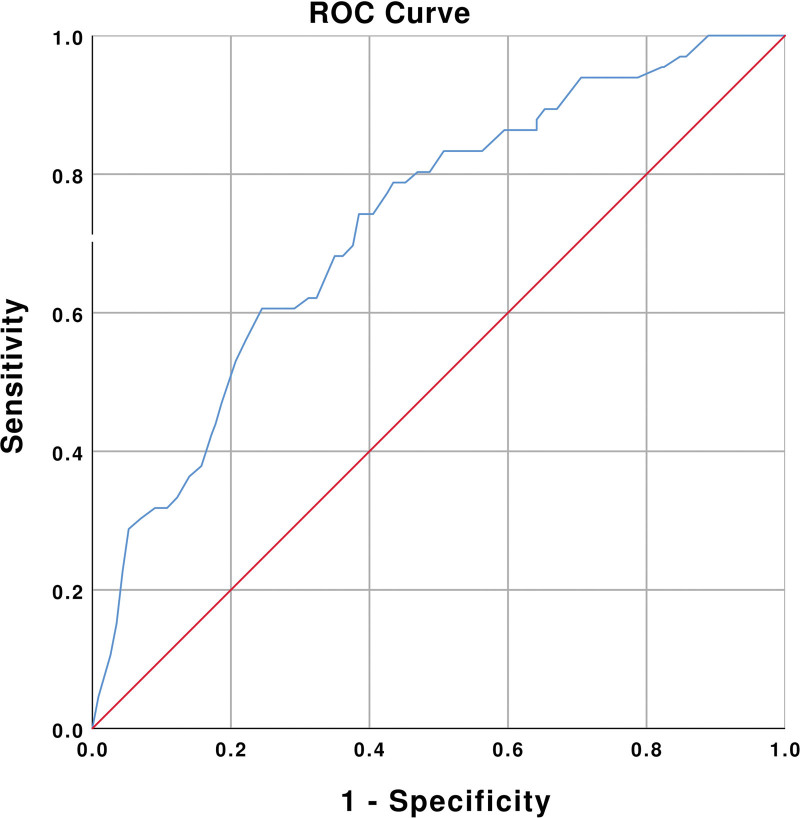
Adjusted AUROC of NLR ≤ 6.5 to predict patient discharge among geriatric patients with influenza infection. AUROC = area under the receiver operating characteristic curve, NLR = neutrophil-to-lymphocyte ratio, ROC = receiver operating characteristic.

AUROC was adjusted for age (*P* < .01), hypertension (*P* < .01), COPD (*P* = .01), and CAD (*P* = .01). The adjusted AUROC for NLR ≤ 6.5 to predict patient discharge was 0.75 with 95% confidence interval (CI) 0.69 to 0.81 (Fig. 2, Table 4). Finally, the performance of NLR ≤ 6.5 to predict patient discharge among geriatric patients with influenza infection showed a sensitivity of 0.74 (95% CI, 0.62–0.84) and a high NPV of 0.92 (95% CI, 0.88–0.95) (Table 5).

**Table 4:** Area under the receiver operating characteristic curve of NLR ≤ 6.5 to predict discharge in geriatric patients with influenza infection.

	AUROC	95% CI	*P* value
NLR ≤ 6.5	0.75	0.69–0.81	<.01

AUROC = area under the receiver operating characteristic curve, CI = confidence interval, NLR = neutrophil to lymphocyte ratio.

**Table 5:** Sensitivity, specificity, negative predictive value, and positive predictive value of NLR ≤ 6.5 to predict discharge in geriatric patients with influenza infection.

	NLR ≤ 6.5
Sensitivity	0.74 (0.62–0.84)
Specificity	0.56 (0.50–0.61)
Negative predictive value	0.92 (0.88–0.95)
Positive predictive value	0.24 (0.22–0.28)

NLR = neutrophil to lymphocyte ratio.

## 4. Discussion

In addition to physiological parameters, this study discovered that the initial NLR level obtained at the ED might play a crucial role in patient disposition, especially patient discharge, among geriatric patients with influenza infection, with an optimal cut-off point of 6.5.

NLR is frequently used to evaluate the severity and outcome of inflammatory diseases, such as sepsis, acute respiratory distress syndrome, and influenza A infection. A study conducted in the ED, including patients with severe sepsis and septic shock, showed that the NLR is associated with a 28-day mortality rate.^[29]^ Another study showed that the NLR is significantly lower in the acute respiratory distress syndrome survivor group than in the mortality group.^[30]^ A retrospective study also concluded that NLR is a potential predictive prognostic biomarker in patients infected with the AIV-human avian influenza A (H7N9) influenza virus.^[31]^ However, none of these studies have mentioned the capability of NLR to predict patient disposition, especially patient discharge, among geriatric patients with influenza infection.

A previous study focusing on the risk factors for mortality in human avian influenza A concluded that a higher mean initial neutrophil count and lower mean initial lymphocyte count are noted in the mortality group than in the survivor group.^[32]^ Similar results were also noted in the current study with a trend of higher neutrophil count and lower lymphocyte count in patients with poor prognosis, including mortality, prolonged hospital stay, ICU, severe inflammatory response (SIRS ≥ 2), and the nondischarged group. As a result, NLR was significantly higher in the prolonged hospital stay group, patients with SIRS ≥ 2, and the nondischarged group, leading to a significantly lower NLR in the discharged group. However, although NLR was also higher in the mortality and ICU groups, it was not statistically significant, with a *P*-value of .95 and .05. These results may be related to the relatively small sample size of the mortality group with patient number = 20 and the ICU group with patient number = 46. A subgroup analysis of an observational cohort study conducted in the ICU also showed similar results, with no statistically significant relationship between NLR and mortality in patients with sepsis.^[33]^

Although the exact role of neutrophils in the pathogenesis of influenza is still ambiguous, the immune system is assumed to be overactivated during severe influenza infection, with substantial cytokine and chemokine release.^[7]^ Cytokines and chemokines can influence many aspects of the immune response, including antiviral defense, hematopoiesis, angiogenesis, and fibrogenesis.^[34]^ A study showed that cytokines, especially serum interleukin-8 levels, are positively correlated with neutrophil counts in patients infected with the influenza A virus.^[30]^ Thus, different levels of neutrophils may indicate the severity of the immune response, including systemic oxidative stress, inflammation, and tissue damage.^[35]^

Discharging patients aged ≥ 65 years may be challenging, as these patients often have complex medical histories and psychosocial issues that lead to longer lengths of hospital stay, increased likelihood of admission,^[36]^ and atypical presentations that require more time and resources to comprehend fully.^[37]^ Therefore, we developed a simple laboratory diagnostic tool to predict patient discharge among elderly patients with influenza infection using NLR, with an optimal cut-off point of 6.5, and adjusted AUROC of 0.75. NLR ≤ 6.5 had a sensitivity of 74% and NPV of 92%. The high NPV level may avoid discharging geriatric patients with influenza infection who need to stay in the hospital and prevent disease deterioration, excess medical costs, and prolonged hospital stay if discharged incorrectly.

Other than predicting mortality in geriatric patients with influenza infection, GID score may also be utilized to predict discharge. Patients with GID score ≤ 1 were considered as low risk (mortality 1.1%), and may consider to discharge the patient.^[21]^ The performance of GID ≤ 1 to predict discharge among geriatric patients with influenza infection revealed both high sensitivity and NPV for 0.95 (95% CI, 0.87–0.99) and 0.97 (95% CI, 0.97–0.99). However, poor specificity and positive predictive value were noted for 0.25 (95% CI, 0.21–0.30) and 0.20 (95% CI, 0.18–0.21) (Table S1, Supplemental Digital Content 1, http://links.lww.com/MD/H108). The adjusted AUROC for both NLR ≤ 6.5 and GID ≤ 1 to discriminate discharge in geriatric patients with influenza infection were identical for 0.754 and 0.750 (Figure S1, Supplemental Digital Content 2, http://links.lww.com/MD/H109). Despite the similarity of the performance of these 2 tools, NLR possesses the advantage of simplicity which required only 2 variables to acquire the result; while GID score required five variables, 2 lab data, 2 medical histories, and 1 vital sign, to complete the calculation.

Discharged elderly patients with influenza infection had a significantly lower percentage of hypertension, COPD, and CAD than nondischarged patients. These results are assumed to be associated with the role of atherogenesis or atherothrombosis of influenza virus, which may trigger preexisting cardiovascular disease by destabilizing vulnerable plaques, resulting in poor prognosis.^[21]^ Furthermore, COPD is one of the most important risk factors for adverse outcomes associated with influenza infection, as the influenza virus itself is associated with COPD exacerbations and results in excess hospital admissions.^[38]^

Despite being the first study to evaluate the use of NLR in predicting patient discharge in geriatric patients with influenza infection, there were limitations to this study. First, some patient data or information may be overlooked due to the retrospective design of this study. Second, selection bias may occur as the current study was conducted in a medical center, and more severe patients with influenza infection may be enrolled. Third, instead of rapid antigen testing, advanced examinations, including reverse transcription-polymerase chain reaction, immunofluorescence assay, or viral culture, should be performed to confirm the diagnosis and subtypes of the influenza infection, as different strains of influenza virus may result in different mortality rates.^[39]^ However, using such expensive and time-consuming advanced examinations during the flu pandemic may result in overcrowded ED and increased medical expenses; instead, it was more practical to use a simple and prompt rapid antigen test. Forth, the results of this study may not be generalizable to other countries because of the single-center setting of this study. Therefore, external validation is needed to verify the results of this study. Fifth, although detailed patients’ discharge condition was not available due to the retrospective setting of this study, none of these 66 discharged patients had 72-hours revisit to the ED, which indicate rather stable condition while discharged from the ED. Finally, the confounding effect of co-bacterial infection on NLR could not be evaluate due to the lack of bacterial culture report. However, evidence of pneumonia and urinary tract infection was used to proxy co-bacterial infection condition, and the result showed no statistically significant difference of NLR between the “evidence of pneumonia and urinary tract infection group” and the “no evidence of pneumonia and urinary tract infection group.”

## 5. Conclusion

When combined with other comorbidities such as hypertension, CAD, and COPD, NLR ≤ 6.5 is a simple and easy to acquire laboratory tool to predict patient discharge during the flu pandemic to avoid ED overcrowding.

## Acknowledgment

We thank Editage for English language editing.

## Author contributions

JCJ, THW, YTT, LFC, and JYC designed and conceived this study. JCJ and YTT wrote the manuscript. JYC performed statistical analyses. LFC and JYC provided professional suggestions. All authors read and approved the final manuscript.

**Conceptualization:** Jing-Cheng Jheng, Yen-Ting Tseng, Te-Hao Wang, Li-Fu Chen, Jui-Yuan Chung.

**Data curation:** Jui-Yuan Chung.

**Formal analysis:** Jui-Yuan Chung.

**Methodology:** Jui-Yuan Chung.

**Resources:** Li-Fu Chen.

**Supervision:** Li-Fu Chen, Jui-Yuan Chung.

**Validation:** Jui-Yuan Chung.

**Writing – original draft:** Jing-Cheng Jheng, Yen-Ting Tseng.

**Writing – review & editing:** Te-Hao Wang, Li-Fu Chen, Jui-Yuan Chung.

## Supplementary Material


