# Supplementary material for: Using neutrophil to lymphocyte ratio to predict discharge among geriatric patients with influenza infection in emergency department
Source: Medicine (Baltimore). 2022 Aug 26;101(34):e30261. doi: 10.1097/MD.0000000000030261 (PMC9410611; doi:10.1097/MD.0000000000030261)
Supplement: Supplementary file 1 [file medi-101-e30261-s001.pdf]

**Table 6.** Sensitivity, specificity, negative predictive value, and positive predictive value of GID ≤ 1 to predict discharge in geriatric patients with influenza infection

| | GID ≤ 1 |
|---|---|
| Sensitivity | 0.95 (0.87–0.99) |
| Specificity | 0.25 (0.21–0.30) |
| Negative predictive value | 0.97 (0.97–0.99) |
| Positive predictive value | 0.20 (0.18–0.21) |

GID, Geriatric Influenza Death; NLR, neutrophil to lymphocyte ratio
