# Supplementary figures and images for: Using neutrophil to lymphocyte ratio to predict discharge among geriatric patients with influenza infection in emergency department
Source: Medicine (Baltimore). 2022 Aug 26;101(34):e30261. doi: 10.1097/MD.0000000000030261 (PMC9410611; doi:10.1097/MD.0000000000030261)

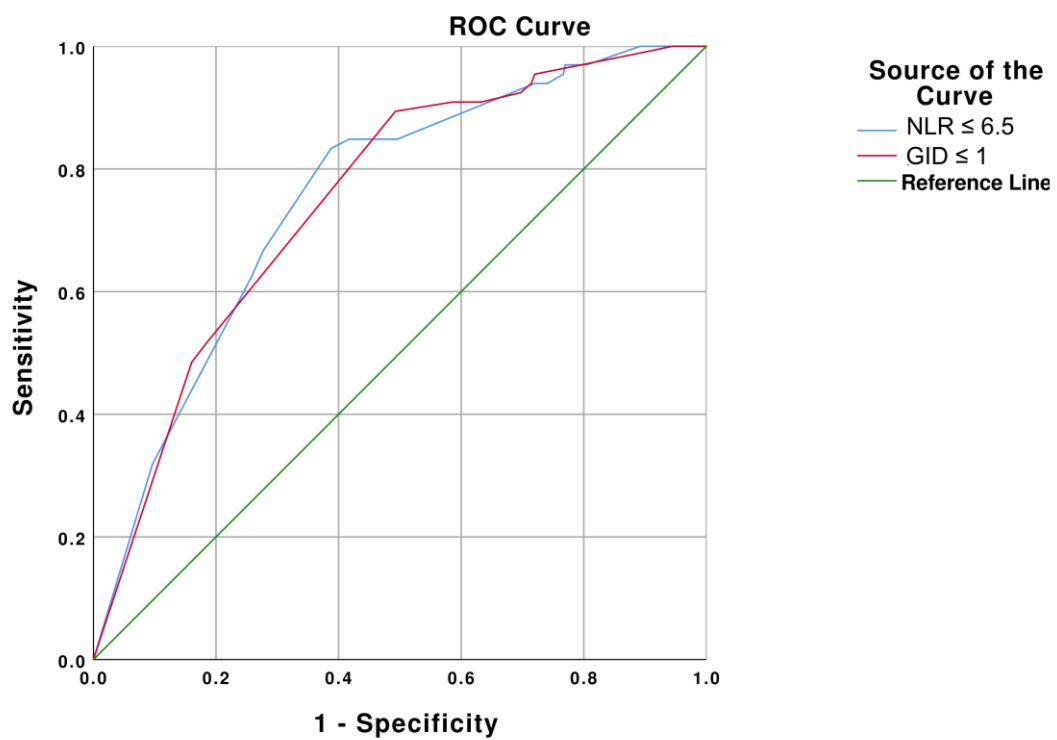

Supplement: Supplementary file 2 [file medi-101-e30261-s002.pdf]
